# The Hour that Never Comes and the Time that Remains

**DOI:** 10.1111/1468-5922.70037

**Published:** 2026-01-17

**Authors:** Daniel Françoli Yago

**Affiliations:** ^1^ São Paulo Brazil

**Keywords:** analytical listening, clinical psychology, mythology, psychic temporality, suspended time, trauma and absence, temporalité psychique, écoute analytique, temps suspendu, traumatisme et absence, mythologie, psychologie clinique, Psychische Zeitlichkeit, analytisches Zuhören, angehaltene Zeit, Trauma und Abwesenheit, Mythologie, klinische Psychologie, temporalità psichica, ascolto analitico, tempo sospeso, trauma e assenza, mitologia, psicologia clinica, психическая темпоральность, аналитическое слушание, остановившееся время, травма и отсутствие, мифология, клиническая психология, temporalidad psíquica, escucha analítica, tiempo suspendido, trauma y ausencia, mitología, psicología clínica, 心灵时间性, 分析性倾听, 悬置的时间, 创伤与缺席, 神话学, 临床心理学

## Abstract

This essay proposes a symbolic and clinical investigation of psychic temporality through two archetypal experiences of time: the hour that never comes and the time that remains. Drawing on analytical psychology, trauma theory and aesthetic philosophy, text explores how certain forms of suffering resist chronological resolution and persist as atmospheres, images or symbolic gestures. The first section examines the temporality of suspended desire and unfulfilled experience—forms of suffering structured not by what happened, but by what never did. The second section explores the aftermath of collapse, when temporality no longer points to the future, but to survival and remainder. Through clinical vignettes, literary references and mythological resonances, the essay argues that analytical listening must not aim to cure time, but to sustain it, especially when psychic life unfolds in waiting, latency or survival. Rather than guiding the patient toward resolution, the analyst may become an Orphic companion: one who listens at the threshold, who stays beside what falls to unfold, and who recognizes the aesthetic, ethical, and political force of suspended time.

## Abstract (fr)

Cet essai propose une investigation symbolique et clinique de la temporalité psychique à travers deux expériences archétypales du temps: l'heure qui ne vient jamais et le temps qui reste. S'appuyant sur la psychologie analytique, la théorie du trauma et la philosophie esthétique, le texte explore comment certaines formes de souffrance résistent à la résolution chronologique et persistent sous forme d'atmosphères, d'images ou de gestes symboliques. La première section examine la temporalité du désir suspendu et de l'expérience non réalisée ‐ des formes de souffrance structurées non pas par ce qui s'est passé, mais plutôt par ce qui ne s'est jamais produit. La deuxième section explore les conséquences de l'effondrement, lorsque la temporalité ne pointe plus vers l'avenir, mais vers la survie et le reste. À travers des vignettes cliniques, des références littéraires et des résonances mythologiques, l'essai soutient que l'écoute analytique ne doit pas viser à guérir le temps, mais à le soutenir, surtout lorsque la vie psychique se déploie dans l'attente, la latence ou la survie. Plutôt que de guider le patient vers une résolution, l'analyste peut devenir un compagnon orphique: celui qui écoute au seuil, qui reste aux côtés de ce qui ne parvient pas à se déployer, et qui reconnaît la force esthétique, éthique et politique du temps suspendu.

## Abstract (de)

In dieser Abhandlung wird eine symbolische und klinische Untersuchung psychischer Zeitlichkeit anhand zweier archetypischer Zeiterfahrungen vorgeschlagen: der Stunde, die nie kommt, und der Zeit, die bleibt. Ausgehend von Analytischer Psychologie, Traumatheorie und ästhetischer Philosophie untersucht der Text, wie sich bestimmte Formen des Leidens einer chronologischen Auflösung widersetzen und als Atmosphären, Bilder oder symbolische Gesten fortbestehen. Der erste Abschnitt untersucht die Zeitlichkeit suspendierter Wünsche und unerfüllter Erfahrungen – Formen des Leidens, die nicht durch das Geschehene, sondern durch das, was nie geschah, geprägt sind. Der zweite Abschnitt erforscht die Folgen des Zusammenbruchs, wenn die Zeitlichkeit nicht mehr auf die Zukunft, sondern auf das Überleben und den Verbleib verweist. Anhand klinischer Vignetten, literarischer Referenzen und mythologischer Anklänge argumentiert der Essay, daß analytisches Zuhören nicht darauf abzielen darf, die Zeit zu heilen, sondern sie aufrechtzuerhalten, insbesondere wenn sich das psychische Leben in Warte‐, Latenz‐ oder Überlebensphasen entfaltet. Anstatt den Patienten zur Lösung zu führen, kann der Analytiker zu einem orphischen Begleiter werden: jemand, der an der Schwelle zuhört, der bei dem bleibt, was sich entfalten soll, und der die ästhetische, ethische und politische Kraft der angehaltenen Zeit erkennt.

## Abstract (it)

Il presente lavoro propone un’esplorazione simbolica e clinica sul tema del tempo psichico attraverso due esperienze archetipiche del tempo: l’ora che non arriva mai e il tempo che si ferma. Facendo riferimento alla psicologia analitica, alle teorie del trauma e alla filosofia estetica, il lavoro esplora come certe forme di sofferenza resistano all’estinzione ed invece permangono come atmosfere, immagini o gesti simbolici. La prima parte considera l’esperienza del tempo nel desiderio sospeso e nell’esperienza inappagata—quelle forme di sofferenza che si strutturano non su ciò che è accaduto, ma su quello che non ha mai avuto luogo. La seconda parte esplora le conseguenze del crollo, quando il tempo non è più proiettato al futuro, ma solo alla sopravvivenza. Con l’aiuto di vignette cliniche, riferimenti letterari e risonanze mitologiche, si afferma che l’ascolto analitico non deve essere finalizzato a trovare una soluzione per il tempo, ma piuttosto a trovare la capacità di sostenerlo, specialmente quando la vita psichica si dispiega nell’attesa, nell’inattività e nella sopravvivenza. Piuttosto che guidare il paziente verso una soluzione, l’analista può divenire un compagno orfico: uno che ascolta sulla soglia, che sta accanto alle cose che accadono e uno che riconosce la forza etica, estetica e politica del tempo sospeso.

## Abstract (ru)

В эссе предпринята попытка символического и клинического исследования психической темпоральности через призму двух архетипических переживаний времени: часа, который никогда не наступает, и застывшего времени. Опираясь на аналитическую психологию, теорию травмы и эстетическую философию, автор исследует неподверженность некоторых форм страдания исцелению временем и их застывание в виде фонового настроения, образов или символических жестов. В первой части рассматривается временной аспект сдерживаемого желания и невыраженного переживания опыта – форм страдания, организованных не вокруг события, а вокруг того, что так и не произошло. Во втором разделе рассматриваются последствия катастрофы, в результате которой время становится направленным не в будущее, а на выживание и застывание. С помощью клинических виньеток, литературных примеров и мифологических аналогий обосновывается идея о том, что аналитическое слушание должно быть ориентировано не на исцеление временем, а на остановившееся время, особенно в случаях, когда психическая жизнь разворачивается вокруг ожидания, застывания или выживании. Вместо того чтобы ориентировать пациента на разрешение проблемы, аналитик может стать орфическим компаньоном: слушать на пороге, присутствовать при том, что начинает разворачиваться, и осознавать эстетическую, этическую и политическую силу остановившегося времени.

## Abstract (es)

Este ensayo propone una investigación simbólica y clínica de la temporalidad psíquica a través de dos experiencias arquetipales del tiempo: la hora que nunca llega y el tiempo que permanece. Basándose en la psicología analítica, la teoría del trauma y la filosofía estética, el texto explora cómo ciertas formas de sufrimiento resisten la resolución en un tiempo cronológico y persisten como atmósferas, imágenes o gestos simbólicos. La primera parte explora la temporalidad del deseo suspendido y la experiencia insatisfecha ‐ formas de sufrimiento estructuradas, no por lo sucedido, sino por lo que nunca sucedió. La segunda parte explora las secuelas del colapso, cuando la temporalidad ya no apunta al futuro, sino a la supervivencia y a lo remanente. A través de viñetas clínicas, referencias literarias y resonancias mitológicas, el ensayo sostiene que la escucha analítica no debe tener como objetivo curar el tiempo, sino sostenerlo, especialmente cuando la vida psíquica se desarrolla en la espera, la latencia o la supervivencia. En lugar de guiar al paciente hacia la resolución, el analista puede devenir en un compañero Órfico: alguien que escucha en el umbral, que permanece al lado de lo que está por desplegarse y que reconoce la fuerza estética, ética y política del tiempo suspendido.

## Abstract (zh)

永不到来的时刻与永不流逝的时间

本文通过两种原型性的时间体验——永不到来的时刻与永不流逝的时间——对心理的时间性经验进行了象征与临床的研究。文章借鉴了分析心理学、创伤理论与美学哲学, 探讨某些形式的痛苦如何抗拒随时间流逝而自然消解, 并以氛围、意象或象征性的姿态而持久存在。文章第一部分考察关于被悬置的欲望与未实现的体验的时间性经验——这类痛苦的结构并非源于已发生之事, 而是源于从未发生之事；第二部分探究崩塌后的余波, 当时间性不再指向未来, 而指向崩塌后的生存与遗留问题。通过临床片段、参考文献与神话之间的回响, 本文主张分析式倾听不应以治愈时间为目标, 而是去维系它——尤其是当心理生活展现为等待、潜伏或生存状态时。分析师不应引导患者走向解决, 而应成为俄耳甫斯式的同行者 : 在阈限处倾听的人, 陪伴在那些未展开之事物的身边, 并认识到悬置的时间所具有的美学、伦理与政治力量。

## Time and How it Collapses

We live besieged by time. Not the time of tides or fruit‐ripening, but a sharp, invasive kind of time: deadlines, metrics and emotional spreadsheets. The time of the world has become measurement, demand, accumulation. The time of the soul, however, resists. And when time suffocates it, the soul falls ill.

In analytical practice, this dissociation comes with painful regularity. There are those who live in a state of waiting for something that never came: recognition, arrival, consecration. Others inhabit the time after collapse, waiting for death: the time of leftovers, of what was never rebuilt but also never vanished. These are lived experiences that defy measurement and do not adhere to the calendar of affective capitalism—a regime in which not only labour and time, but also emotions, relationships, and inner life are formatted by demands of productivity, optimization, and self‐performance. In such a context, even grief, latency, and ambivalence are pressured to “evolve” into value: healing must be quick, suffering must be meaningful, and all desire must be actionable. Yet there are psychic experiences that do not yield to this logic. They resist conversion into goals, and thus require another kind of temporality that analysis must learn to accompany.

Carl Jung (2011), recounting his own journey in *Memories, Dreams, Reflections*, writes: “Everything seemed suspended in time; there was no longer any relation to the present (…) the experience of being outside of time gave me a sense of absolute freedom” (p. 309). This account, situated in a visionary experience, points to a dimension of time not measured by clocks, but by symbolic intensity. A psychic time, where what matters is not when something happened, but how much it still acts.

Walter Benjamin (2006), an important philosopher of the Frankfurt School known for its critique of modernity and instrumental reason, names this logic in another key. In his fifth thesis *On the Concept of History*, he speaks of historical time as non‐continuous, but interrupted—a time full of shards, where moments open up as cracks: “The past can be seized only as an image which flashes up at the instant it can be recognized and is never seen again.”

In this essay, this glimpse that refuses the progressive continuity of history is kin to two categories we will explore: “the hour that never comes” and “the time that remains”.

The first concerns the time of suspended desire, of the event that never occurred, of the trauma that froze the flow of experience. The second refers to the time after the fall, to what still vibrates after collapse, as echo, as remainder, as fold, as fidelity to which no longer returns. There are times that do not serve us. Times that refuse to function. Our inquiry is into the times of the soul, in the Jungian sense. These are expressions of an inner temporality that does not conform to external rhythms but also resists pure chaos. They are forms of psychic life that unfold as images, affects, compulsions, silent rituals, or, in other words, they are symbolic necessities.

The soul’s time, therefore, is spiral. It does not seek to arrive anywhere, but to reach the depths, and perhaps sings there, like Orpheus. By “soul”—by no means an obvious word—I refer not to a metaphysical entity but to what James Hillman (1996) describes as the poetic basis of mind: a mode of seeing that deepens experience, insists on image, and resists literalism. Soul is not a substance, but a perspective—one that finds meaning in repetition and in shadow. Its temporality is not progressive, but recursive; not strictly teleological, but also imaginal. In this context, to say the soul’s time is spiral is to affirm that it returns, circles and fold back on itself—a way of descending further into meaning. This difference between outer and inner time is more than a conflict of agendas: it is the very terrain of Jungian thought. How to listen to a subject whose suffering cannot be localized in the now, but occurs in another time, one that insists, repeats, or remains? How not to push this time to resolve itself, to adjust, to “move on”?

This reflection is only possible because I have stood on the shoulders of giants who, before me, thought deeply about the multiple rhythms of psychic time. In *Time and Free Will* (1910/2001), French philosopher Henri Bergson proposed a distinction between clock time—homogeneus, quantitative and spatialized—and real duration—an inner flow that is qualitative, continuous and irreducibly lived. Jung not only knew these formulations, but integrated aspects of them into the conceptual body of analytical psychology. Pete Gunter (1982), in his study of the relation between Bergson and Jung, shows how the concept of intuition—central to Jung’s ideas since since *Psychological Types* (1921/1971)—has roots in Bergson’s idea of direct, non‐discursive knowledge. From a Jungian perspective, the psyche is not merely subject to chronological time; it pulses according to its own intuitive and imaginal rhythms. The time of the soul—the one I seek to listen here—is shaped in part by the notion of duration as a living, symbolic flow, in which past, present and future are not distinguished by sucession, but by intensity of presence.

I also inherit, with critical gratitude, the distinction between Chronos and Kairos—two modes of time which, in Greek tradition and later philosophy, contrast the linear chronology of events with the meaningful irruption of opportune moment. Yvette Weiner (1996), in a seminal article, proposes that the consulting room is precisely the place where these two times meet and clash: Chronos organizes the frame, the commitment, the routine; Kairos, on the other hand, evokes moments of symbolic opening and transformative listening. Rather than contradict this division, I aim to extend it. The hour that never comes and the time that remains may not be direct expressions of either Chronos or Kairos, but they emerge within the field of tension between them. They are not a third time, but perhaps a residue, a distortion, or a fold produced by their clash. An unlived time that repeats itself, and a surviving time that drags on, fits neither category entirely. These temporalities, unfolding in the atmosphere of analysis, may stem from the friction between the time of structure and the time of revelation—and it is within this friction that the symbolic spiral I seek to listen to begins to take form. In this sense, time may be less a measurable sequence than a fiction of orientation—what Randy Charlton (1997) calls a symbolic construction that shapes psychic life much like the internal object: it allows experience to cohere around absence, latency or desire.

This essay is born from encounters with patients whose suffering does not obey the calendar. It sets out to investigate how psychic time manifests in experiences of suffering that do not heal with time, and proposes an analytical listening not aimed at resolving time, but at sustaining it in its most Orphic form: suspended and atmospheric. To traverse the forms of time that fail is to imagine a listening capable of inhabiting those failures without seeking to close them. In the following sections, we will explore these two analytical forms of time: the hour that never comes, and later, the time that remains.

The path is open. May time find us well.

## The Hour that Never Comes

“I think I’m behind in life.” Few phrases appear so frequently, and with such weight, at the beginning of an analysis. The patient arrives and describes themselves as someone who missed a train no one saw depart. They know they’re alive, but don’t feel in motion.

The delay here is not literal, but symbolic. It’s as if the future is always one step ahead, and the present drags behind, trying to catch up. But this race isn’t real, it is lived internally. What is delayed is not the résumé, the wedding, or the bills: it is meaning.

Jung (1921/1971, para. 761), in discussing psychological types and the process of ego differentiation, already recognized that the psyche has its own temporality, and that following external timeframes can be an act of violence. In *Psychological Types*, he writes: “the goal of life is not outward success, but the inward realization of the personality.” When that realization fails to begin or feels interrupted, suffering emerges—the sense that something should have happened, but didn’t. It’s the feeling of being out of sync, as if others are dancing to an inaudible song, while the subject stands frozen at the edge of the ballroom.

This time—the hour that never comes—is composed of unfulfilled promises, rites of passage that never took place and images of the future that never found a body. The person doesn’t quite know what they’ve lost, but feels indebted to something never lived.

It’s not just trauma at stake. It’s the non‐event that weights on them: an absence that has taken form. The complex, in this key, is not merely a painful memory—it is a paralyzed psychic field, like soil where nothing has grown, yet whose furrows still mark the earth. This delay is not always undesired. Sometimes, as the Jungian analyst Verena Kast (2019) suggests, it is maintained by the soul itself—as if it refuses to follow a path it senses is false, as if it were saying: “I will not hurry to fulfill a story that is not mine.”

There is wisdom in the non‐happening. And that’s why analytical listening must not attempt to accelerate time. There are patients whose symbolic task is precisely to inhabit that in‐between as fidelity to a desire or an image. They are delayed because they do not wish to betray themselves. Perhaps, as Hillman (1996) writes, the ego seeks progress, but the soul seeks something else: we must call the soul back from its literal exile, where it has been forced to serve improvement. Symbolic delay, then, may be the soul’s way of refusing to be domesticated, a refusal to move forward just because that is what is expected; a refusal to enter the rhythm of the world in order to preserve its own.

It is not always what happened that traumatizes. Sometimes, it is what never happened: the touch that never occurred, the word left unsaid, the justice that came too late, the recognition that never arrived. The event that never fulfilled itself, and yet, shaped a life. The unfulfilled, delayed hour is one of the most subtle and profound forms of psychic suffering: the non‐happening that becomes memory, the void that gains contour. Trauma without an event. The mark of an absence. Absent mothers and fathers become the most present figures in someone’s life precisely because—being spectral—they can potentially be everywhere, as the Jungian analyst Susan Schwartz (2023) once stated.

The literary and trauma theorist Cathy Caruth (1996) proposes that trauma is not only what wounds the subject at the moment it occurs. It is also what happens too late, when there is no longer time to metabolize. Trauma, she writes, “is not simply a wound inflicted by a violent event in the past, but an impact that persists, as a ceaseless return, in the present moment” (p. 11). In the case of what did not happen, the logic is similar: what should have been lived returns not as memory, but as suspended time—a waiting that turns into compulsion, a desire transformed into symptom. In analytical work, this suspended time often emerges disturbingly. The subject repeats scenarios of abandonment, helplessness, “almosts”: projects that don’t move forward, dreams that seem to carry, from the start, their own impossibility.

This idea—which traces back to early psychoanalytic and post‐Jungian formulations, notably in the works of Michael Fordham and Mervin Glasser—finds a powerful unfolding in the work of Kalsched (1996), who explores early trauma as a deep psychic rupture requiring dissociative defences often animated by archetypal forces. He describes what he calls the “personal spirit”, the most sensitive and imaginative part of the child, as fragmented and protected by highly organized internal symbolic structures. These defences, though they preserve psychic life, trap the subject in a dissociated temporality, where the trauma remains “frozen” as image.

In this case, the time of the trauma of what never happened is not a broken line, but a vicious circle. The present revolves around what did not occur. Each new attempt at life returns to the same edge, as if the psyche were caught in a fold. Here, we are deeply inspired by Leda Martins (2025), one of Brazil’s foremost thinkers, who offers a powerful epistemic key to this experience through her notion of spiral time, in contrast to the linear temporality of European colonialism. Drawing from Afro‐diasporic traditions—rooted in the lived experience of displacement and ancestral memory rituals practiced by Black communities across the globe, particularly as a result of the transatlantic slave trade—her work proposes a temporality in which the past does not recede, but insists as a recursive unfolding of what remains unresolved, rather than a return to the same. Spiral time is about reverberation: each return reactivates a wound that was never allowed to close, and in doing so, keeps the past alive within the present. This temporal logic ressonates with the psychic experience of trauma, which often unfolds not in the chronology of events, but in the ritual recurrence of what could not be integrated. She writes: “Spiral time renders the past a living presence, and the future an updating of what could not be” (Martins, 2025, p. 14).

This updating of what never was is precisely what we witness in patients who live in the hour that never comes. It is not merely postponement, but the continual elaboration of an absence, a wound that does not heal because it never bled. It just stayed there. Silent. Waiting. This kind of trauma requires a radically different form of listening. It is not about locating an event, but recognizing an absence. And more: recognizing that this absence organizes subjectivity. The absence becomes central. Waiting becomes structure. And time, instead of flowing, orbits around what never arrived.

Often, this field of non‐happening is populated by recurring images: thresholds, almosts, the unfinished. A kiss not given. An invitation that never came. An “almost love”. An “almost recognition”. As if the psyche were perpetually staging the moment just before life. Hillman (1996) might say the soul is trying to shape itself around the void—to give symbolic body to that which never had time to become image. Here, analysis does not need to “heal” the absence, but to listen to it as form.

Suely Rolnik (2018), Brazilian psychoanalyst and Deleuzian cultural theorist, speaks of the need to create psychic spaces that do not accelerate becoming, but that welcome latency—that which has not yet actualized, that which needs more time to become sensible. She writes: “Life’s pulsation is not linear or constant. It needs silence, pause, waiting” (p. 46). Suspended trauma, then, is also latency, a pain that does not explode but hovers, an expectation that refuses to die and, thus, continues to orbit us.

Perhaps the most cruel form of time is this: the one that insists but does not advance; the time that does not pass because it never began. The hour that never comes because it does not know how to arrive. Because no one called it by name. Because no one stayed long enough to listen. And that is what analysis can offer: a space where absence is listened to, where what never was might finally take shape—even if that shape is silence. Even if it is an image. A shadow. A broken song, but one that still echoes.

Not all waiting is the same. The hour that never comes can be lived in two very distinct, and sometimes overlapping, ways. Some postpone the future as a symbolic gesture of the soul, a refusal to enter the world’s time. Others are unable to cross the present because it accelerates, thickens, multiplies, and paradoxically paralyzes. One is a time frozen by too much expectation; the other, by too much fear.

In the first form, postponement is a refusal to move forward in order to protect something essential: the refusal to become an adult, the denial of a call that feels false, the fidelity to an old sorrow. These are people who are “late” not because to failure, but due to an invisible loyalty to an image, a grief, a broken promise. The future is there, but they refuse to enter it. This waiting protects. This gesture finds a parallel in the myth of Demeter, the goddess of fertility, who, when her daughter Persephone is abducted by Hades, suspends the seasons. The world’s time stops: no seed sprouts, no flower blooms. Demeter refuses to continue the cycle of life until what was taken from her is returned. Postponement becomes both a form of mourning and a symbolic power. She is the one who chooses not to let time move forward until her grief is acknowledged. In analysis, there are patients who, like Demeter, withhold time as symbolic resistance. They refuse to “move on” until a pain is heard. The soul is saying: “I do not accept the logic of overcoming”. Waiting here is a gesture of psychic dignity—a grief refusing to be assimilated, a childhood refusing to be forgotten.

But there is a second face to the hour that never comes—one born from anxiety. A time that does not refuse to move forward: the subject wants to proceed, wants to decide, wants to act—but is stuck in a dense, hyperaccelerated present, where nothing stabilizes enough to become a choice. It is the time Byung‐Chul Han (2012/2015), South Korean‐German philosopher, describes in *The Transparency Society* as the time of hyperexposure and continuous performance. There are no intervals, no backstage. Everything is visible, everything demanded. In this model, time does not pass, it only loops. The philosopher writes: “The transparency society is a society of absolute present, in which the difference between future and past disappears” (p. 45). It is the time of anxiety: the now endlessly renewed, endlessly insufficient. Here, the myth that emerges is that of Sisyphus, condemned by the gods to push a stone up a mountain, only to watch it roll down again, endlessly. There is no progress. Only repetition. Effort without movement. Time without becoming. In analytical practice, this time appears in patients who live in a permanent state of obsessive vigilance. They wake up already feeling behind. They go to sleep feeling guilty. In their accounts of the week, they cannot say when the days began or ended. They feel that time devours them. It’s like being in a car with the engine running but never shifting gears.

Distinguishing these two forms of suspension—the soul that postpones and the anxiety that paralyzes—is fundamental. One asks for listening; the other, for deceleration. One leans toward myth; the other, toward collapse.

But it is not always easy to distinguish them, because often, they intertwine. The subject who delays out of fear of trauma may repeat out of fidelity to desire. The soul is ambiguous. Psychic temporality, even more so. Rolnik (2018), reflecting on the effects of neoliberalism on subjectivity, speaks of a vital pulsation that is neutralized by an excess of updates. She writes: “the contemporary power system operates by neutralizing life’s potency through the imposition of permanent updating, preventing the necessary time for difference to germinate” (p. 62).

Postponement, in this context, may be the last gesture of freedom, the refusal to be colonized by the time of capital, the soul’s “no” to the time of productivity. Perhaps, deep down, the hour that never comes is always the same hour—the same field of tension between not wanting and not being able, between the gesture of waiting and the panic of incapacity. And analysis must become the place where time can be heard in its full complexity.

The soul speaks in images. And when the hour does not come, it is common for the psyche to begin producing symbolic forms of this delay: dreams, fantasies, recurring urges—scenarios that repeat as if begging for interpretation. though perhaps all they need is to be heard. These are images of thresholds, of interrupted crossings, of incomplete motion: doors that won’t open, stairways that lead nowhere, endless corridors in ancient houses, locked rooms, elevators that never arrive, passages that never actualize. The subject dreams that they are always on the verge of approaching, but never crossing. The anguish does not arise from what is on the other side, but from the failure to cross.

These images carry millennia of symbolic weight. In myth, crossing is always crucial: crossing the temple threshold, descending into the underworld and passing its gate of despair, stepping through the mirror. When that does not happen, the myth collapses. In art, this liminal space is vast and fertile. Caspar David Friedrich, in his celebrated *Monk by the Sea* (1808), depicts a solitary figure facing a sea without horizon. There is no action, only imminence. The open space becomes emptiness, and suspended time. Nothing happens, but everything is saturated with potential. The image is nearly unbearable, because it offers no resolution. In cinema, one of the purest expressions of this image appears in the opening sequence of *Persona* (1966), by Ingmar Bergman. The screen flickers with disjointed images—among them, a boy who touches a translucent screen with his fingers, as if trying to reach someone on the other side. But there is no reciprocity. The other side remains opaque.

This type of image is not merely illustrative: it is the very symptom of a soul‐in‐waiting.

In the analytical setting, patients bring such images as if confessing a delusion. But the attentive analyst knows that a symbolic vocabulary is being forged there. The image comes as a way to give body to what cannot yet be said. Sometimes, it is the only narrative possible. For example, there is C., who is 29 and arrived saying, “life stopped on a single frame.” They speak of opportunities that vanish before becoming real, of affections that form like clouds, but never rain. They frequently dream of climbing narrow, spiral staircases toward doors that will not open, or that do open, only to reveal more stairs. An inner Escherian labyrinth. Once, they described a dream in which they descended to a subterranean library and found a book with their name.

Rather than rushing to interpret, listening must sustain the time of the image. Let it speak, or let it remain silent. In time, they began to draw those stairs. One day, they brought a sketch where all the staircases were suspended in mid‐air, not touching the ground—“like the beginning of the manga *Berserk*”, a scene I later found in the third volume of Kentaro Miura’s brilliant series (2020). “It’s like I live in the path,” they said, “but the ground still feels more threatening than the demons that live there.” These images, though seemingly stagnant, carry symbolic movement. The psychic delay is inscribed within them—the unconscious as an ever‐boiling cauldron. The unconscious in its purest form: imaginal, repetitive, metaphorical.

It is important to remember that many of these images are not “symptoms” in the psychopathological sense. They are poetic forms of waiting. The stair leads nowhere because there is nowhere to go, at least not yet. The door does not open because it is not time.

Jung (1944/1968, para. 297), in *Psychology and Alchemy*, speaks of the image as a bridge between consciousness and the unconscious. He writes: “The images of the unconscious are, so to speak, representations of the process of psychic transformation.” So that these liminal images are not static: they are intermediaries of becoming. They announce a time that has not yet arrived, but already glimmers. They are how the soul refuses to give up. Analysis, at such moments, must not accelerate the passage, but dwell at the threshold alongside the patient. Listen to the stairway. Honour the door. Wait within them, side by side, for the time that has not yet opened.

As it was said, not all waiting is passive. Some forms of waiting are fidelity, interior gestures of those who refuse to accept just anything in place of what was once desired. A kind of “yes” to the original desire, even if it cannot, for now, be fulfilled.

In analysis, it is common for waiting to be experienced with guilt. We think we’re stuck—but maybe part of us wants to stay exactly there: still unread, incomplete. Because something is still there, like an image, a promise, a love, a vocation, that cannot be replaced. After all, the soul is not interested in solutions, but in depth. And that is why it waits: because it knows that rushing would often be a betrayal of the essential. Waiting, then, becomes not emptiness, but a form of care for the image that has not yet matured. It is like not picking the fruit before it is ripe.

In myth, this dimension appears clearly in figures such as Penelope, who for 20 years weaves and unweaves the same shroud, awaiting the return of Odysseus. Suitors pressure her. The kingdom beckons. But she remains faithful to what she chose. Waiting becomes ritual, creation, textile art—and, above all, affirmation. In analysis, there are patients who make a similar gesture. They refuse to love “just anyone”, to work in “just any job”, to live “just anyhow”. Even if they suffer for it. Even if they feel the exhaustion of the postponed promise. Sometimes, waiting is stubborn—it’s the psyche refusing to bury an old dream, even when the world says: let go.

There is no resignation here, only knowing. Waiting is not an absence of events. Sometimes it’s a strategy, a stubborn temporality, a way of being with what is not yet, but already has a name. Analysis must be a place where this kind of waiting is not treated as pathology, nor as a planning failure or lack of ambition, but as a gesture of the soul that refuses to be trained in the time of things.

Of course, there are lots of sterile waitings. Clinging to the past can become a prison. Idealization can suffocate the real. But it is also true that many of our deepest desires cannot withstand premature realization. They require symbolic gestation, unconscious maturation—a different kind of time. Let us remember Orpheus, who descends into Hades not to seize Eurydice by force, but to sing her absence. His journey is active waiting in the dark. And that is what Orphic listening proposes: not to accelerate the story, but to wait for the time of the soul, even if it is dark, slow, sticky, oblique.

Waiting may not be paralysis, but a form of relationship with desire. And the hour that never comes does not need to be rushed. It can be listened to. And that is where analysis distinguishes itself from other forms of care—even if it finds no resolution, even if it remains inconclusive. Nothing seems to happen. Yet something always trembles with potential. This is the time when the psyche speaks the most. The analyst does not interpret on impulse, but listens as someone who sits with their patient at the bank of a river that has not yet reached the sea. Listening to suspended time demands an ethics—and an aesthetics, too. It requires what Rolnik (2018) calls a “vibratile field”, a sensitive state that welcomes what has not yet taken form. Analytical listening must be done with the whole body, in a state of loving attention to the forces that have not yet actualized. This means that listening to suspended time does not seek answers, but presence.

By allowing the hour that never comes to be heard as it is—dense, ambiguous, empty of events, yet full of latent meaning—analysis transforms delay into symbolic time, and waiting into a creative detour. The time of analysis, at this point, is the time of an unhurried crossing. The hour has not yet arrived, but the simple fact that there is listening already alters the passage of time.

And that is enough. Sometimes, enough.

## The Time that Remains

Time does not stop, but it can collapse. In fact, the fall doesn’t always end when the body crashes to the ground. Some patients keep moving, taking measured steps, meeting obligations, yet they carry a silent collapse within. Analysis does not necessarily detect a specific trauma, nor a concrete rupture. What is heard is something more diffuse: the sense that time no longer moves forward. In the analytical work, this is fact: “I no longer know what comes next,” “my life has stopped,” “it’s as if I’m still walking, but inside I’ve already ended.”

These phrases do not describe the past. It is the time that remains: what lingers after the collapse of any expectation of future. It is not experienced as transition between two moments, but as the residue of history, as the echo of a distorted timeline. The soul remains in motion, but without horizon, as if everything had already happened.

Peter Pál Pelbart (2000), Hungarian‐Brazilian philosopher, in *O Tempo Não‐Reconciliado* [The Unreconciled Time], explores time as nonlinear multiplicity. Instead of an arrow toward a telos—the Jungian model—we encounter a labyrinth of simultaneous presences, variations, and discontinuities. He writes:

Instead of a timeline, we have a tangle of time; instead of a flow of time, a mass of time emerges; rather than a river of time, a labyrinth of time. Not a circle of time, but a vortex; no longer an order of time, but infinite variation; not even a form of time, but an informal, plastic time. With this, we are undoubtedly closer to a time of hallucination than to a consciousness of time. (Pál Pelbart, 2000, p. XXI)

This idea of time as vortex does not lead to reconciliation, but to the impossibility of closure—the experience of a saturated present that connects neither linearly to past nor future. When someone lives after the fall, they inhabit this zone where chronologies fragment and time no longer points forward. There is no longer narrative, no teleology. There is only a dense substance, where events no longer arrange themselves. Everything vibrates but nothing progresses. And this is not resolved by explanation.

Mark Fisher (2009), British cultural theorist, describes this rupture as the cancellation of the future. It is not merely a psyche that has lost the ability to imagine another time: the future has been emptied, aestheticized, neutralized. After all, “it is easier to imagine the end of the world than the end of capitalism” (p. 2). This now‐proverbial phrase masks an analytical impact still underestimated: it speaks not only of politics, but of soul. Capitalism does not merely dominate the present—it colonizes the imagination of time itself, making the future inconceivable as anything other than more of the same. Tomorrow becomes nothing but an endlessly remixed version of today. It is not the past that is repeating, it is that the future has been cancelled.

This experience is the stiffening of temporality. The subject no longer dreams because they no longer believe in a symbolic space where dreams can find realization. Time becomes a treadmill: continuous, accelerated, yet immobile.

This is precisely what Han (2016/2018) observes from a different angle. In *The Expulsion of the Other*, he claims we live under a regime of narcissistic repetition of the same, where everything is updated, shared, visible, but nothing truly happens. Difference, once the founding force of time and the opening to the other, is replaced by a continuous, tautological, undifferentiated now: “The acceleration of time does not generate future, but repetitions. The new does not arrive; only more of the same” (Han, p. 19). The result is a peculiar form of collapse: not sudden catastrophe, but hyperactive stagnation.

Perhaps Pelbart, Fisher, and Han are each addressing different facets of the same temporal wound: the first, the collapse of symbolic linearity; the second, the dissolution of the future’s imaginative power; the third, the saturation of the present as a form of blockage. It is in this convergence that one hears, in analysis, the subject who cannot desire forward because there is no longer an internal or external time where it might inscribe itself. In this context, melancholy is no longer only mourning for what was lost—it becomes the perception that there is nothing left to lose, because there is no more future. The subject lives in leftover time, disbelieving in metamorphosis.

In Hillman’s (2024) essays on depression and melancholy, the latter does not appear as a pathology to be overcome, but as an atmospheric mood of the soul that permeates both the individual and the world. For him, personal depression is inseparable from the depression of the anima mundi, a sign that something in the world has lost symbolic resonance. When melancholy ceases to be an image and becomes literalized in its pathological form, it loses its potential to reveal, imagine and transform. In this shift from imaginal to the literal, clinical work risks adressing only the symptom, without to what melancholy is asking to express as an articulation of the soul.

Anne Dufourmantelle (2011), French psychoanalyst and philosopher, in *Éloge du Risque*, proposes an ethics that refuses the imperative of overcoming. Instead of demanding that pain be turned into virtue, or that wounds become lessons, she invites us to a form of listening that is truly hospitable—even to what does not settle, to what remains excessive. For her, traumatic time is not redeemed. It insists. And perhaps it can only be welcomed by a presence that does not seek to explain it, nor dissolve it, but simply to accompany its intermittent eruption. In *La Sauvagerie Maternelle* (2019), this radical listening gains even sharper contours: trauma is not merely an event to be deciphered, but a kind of primal force that interrupts organized time, inaugurating another temporal regime. Dufourmantelle does not speak of cure. She speaks of shelter. To welcome this wild time is not to domesticate its logic, but to let oneself be affected by it.

It is in this sense that analysis can operate not as a reconstruction of the timeline, but as the creation of a space in which linearity is no longer required. The time that remains—this time after the fall, without project and without promise—need not be transformed into narrative. It can simply be heard, like someone listening to the creaking of an old house or the wind striking ruins.

No analyst has the power to restore futures or to carry the map of listening. The idea here is to remain beside that which does not move. To believe that even after the end, the soul still breathes. There are those who still wait, and those who have already stopped waiting, not out of meaning, but simply because there is a body, a residue of time in which one is inscribed. After the fall, time does not vanish: it remains. And that remainder is what many subjects live: a present that is ruin, a kind of elongated now, emptied of project, yet filled with survival. In analysis, this time appears as a form of exhausted presence. The patient shows up, speaks, works, even laughs at some of my jokes, yet carries a fundamental disconnection from the future. Tomorrow is the mute continuation of today. And today is a pile of rubble. “I’m doing what I can,” “I’m getting by,” “that’s all there was for today,” “I’m dragging myself along.” Phrases like these signal something more nuanced than depression: a life that hasn’t ended but is no longer grounded in promise.

The present, then, ceases to be a bridge and becomes scorched earth.

And this is what also appears in the analysis of patients who live a present shaped by traumatic inheritances, structural exclusions, a fatigue that only the history of the world can explain. For them, to live is to carry the Sisyphus stone. Listening, in this scenario, seeks symbolic dignity for existence as it is.

A., 53, came to the consulting room without urgency or despair or diagnosis. He wasn’t seeking help or salvation. He simply said something in his life has ended, although nothing in particular had begun in its place. He was just over 50, had taught literature for more than two decades, but had been on leave since his father’s death—a father he had cared for in the final years, in a small, quiet apartment. After the funeral, he never returned to work. It wasn’t “exactly sadness,” but “time had lost its folds.” “I still wake up,” he said once, “but the days no longer come with me.” There was no visible distress, no disorder. He dressed with care, wore perfume, kept to schedules, asked thoughful questions. Yet around him, everything seemed emptied of sequence, as if events had lost the ability to follow one another. He spoke of his life with precision but without gravity, like someone recounting a dream from which they have long since awakened. In his speech, the past was not remote, only irrelevant; the future was not absent, only unnecessary. At times he said he remained alive out of distraction. At others, because no one had called him back. He never asked for advice. He simply kept returning—even to my surprise—week after week, as if waiting for something in him, or in us both, to find the rhythm again. His existence was not exactly sad, but it happened in a lowered key. He expected nothing, refused nothing, simply remained there, inhabiting a time that no longer belonged to anyone, yet still belonged to him, in a paradoxical way.

The imaginal figure that constellates here is not the hero, nor the orphan, nor the pilgrim. It is the wanderer among ruins—something between the Tarot Hermit and Benjamin (2006)’s Angelus Novus—the one who knows the building has collapsed, yet continues to walk among the stones, collecting useless objects, repairing cracks that perhaps no one else will ever notice. This kind of time cannot be healed.

The time that remains implies living not as someone who chooses, but as someone who endures—not because there is promise or desire, but because time still pulses, even when we no longer know where it goes. Survival is not merely what is left of life. In my analytical work, I have met patients who did not seem to be fully alive, in the complete sense. But they were not dead either. They were in between: between a story that no longer speaks and a future that refuses to reveal itself. As if life continued through symbolic automatism, psychic inertia. Affects repeat. Gestures persist. But something is broken, something hollowed out.

This experience lies at the heart of Jung’s *Red Book* (2009). After his break with Freud, Jung went through a deep psychic and emotional collapse. He lost the ground of theory, identity and narrative. But he did not seek immediate meaning: he descended, and began to listen to images arising from the depths. There was no project. What there was, was a time that does not advance but expands inwardly, like a spiral: “The spirit of the times wanted to force me to say who I was. But I did not know. So I went down into the darkness” (Jung, 2009, p. 229). That descent is a form of symbolic survival: to go on living in the absence of explicit meaning, trusting that something still pulses, even in the dark.

Analysis often returns to that point: when narrative disappears, the only thing left is to listen to what insists. Michel Foucault (1966), French philosopher, also recognizes this experience. He speaks of language as limit: there are things that cannot be said and yet still insist on being said. The subject of the outside is one who lives on the edge of language, in the cracks of the sayable. This outside is not exclusion—it is the survival of that which cannot be named, but which still resounds.

Survival, in this sense, is not empty. It has texture. It has noise. It is the field in which what was not fully lived still pulses, like an echo. Time, here, does not point toward the future—it resists in the reverberation of the past. This landscape of survival may find its most potent image in the work of Anselm Kiefer. His burned canvases, marked by lead, ash and charred wood, depict a time that has passed but not vanished. Paintings made of rubble. Thick layers of matter. Times that do not resolve into clear image, but remain like open wounds, beautifully suffocating. To look at a Kiefer painting is like listening to a patient in survival mode: everything seems undone, and yet there is an insistence, a dense, unbargainable presence. The analyst, in such moments, need not understand. Survival is the time of one who expects nothing anymore, but still hears the noise of the living.

T. was 51 when she began analysis. Just like A., she had mourning issues. She arrived with a restrained voice, but a clear phrase: “I don’t have a specific reason to be here. But I also have no reason not to be.” She had lost her daughter 2 years prior, in a car accident—and her job, after 27 years, 6 months after the death. She insisted it wasn’t depression. She woke up, ate, went shopping, talked with friends. Took no medication. No longer cried. But she also no longer dreamed. “It’s like my life has turned into a landfill. Things are there. But I don’t know what they’re for.” T. did not say “my daughter died.” She said: “she didn’t come back.” She spoke of time as one speaks of a place that ceased to exist. “Since then, everything has tasted of after.” What moved me most about T. was not grief, but the silent presence of someone who continues. She made a point of showing up for sessions even when she had nothing to say. We shared many silences together. “Are you just waiting for time to pass?” I asked. To which she replied, “No. I just don’t want to throw away what’s still here.”

Until one day she told me about a recurring dream. She’s walking along a dirt road. The ground is red and dry. On the roadside, there are pieces of houses: windows, tiles, fragments of wall, as if someone had demolished the world and left everything there. But the strangest thing is that she feels no fear. No sadness either. In the dream, she walks calmly. She picks things up from the ground. Sometimes she sits. Sometimes she continues walking. In the end, she always wakes up with the sentence: “There’s nowhere left to get to, but I’ll keep walking.” Her analysis with me didn’t bring back a dream of the future, but perhaps it gave her a space to live without one. In one of the last sessions of the year, T. said something of great value: “I think I’m trying to learn to live as if the world had ended, but I was still in it.” There was no lament in that phrase, only subterranean courage. And also an echo, like the sound of footsteps among ruins, which both of us heard together in the space of analysis.

The collapse of lived time, as seen in A. and T.’s cases, can be understood not merely as a consequence of trauma, but as a specific condition of the individuation process. When the linearity of time breaks down—through loss, the suspension of dreams, or the failure of continuity projects—what opens up is not emptiness, but a field in which the soul’s temporality may finally emerge. This is a spiraling time, laden with images that follow not the logic of progress, but that of fertile repetition, suspended listening, and ripening delay. In this sense, individuation is not a return to normalcy, nor a form of functional adaptation, but a symbolic traversal that becomes possible only when the ego yields to the irregular rhythms of the psyche. Analysis does not aim to reconstruct lost time but to create a ground where what remains—ruin, fragment, silence, leftover—can be held as a form of presence. As Hillman intuited, and Jung before him, it is at the margins of normative time that the soul begins to speak, and it is in this interval that analytic work takes place

So, if what remains can, indeed, be silence, it can also be gesture. We know that the time that remains is not merely what is left after the fall. It is what insists on remaining, even without purpose. Ruin is one of its strongest images. So is the fragment. But above all, what insists on remaining may be the possibility of creating with what still exists. It is not a time for rebuilding, but a time for groping among the rubble. In order to better grasp this idea, we must go beyond concepts and evoke certain artistic sensibilities that offer an alternative path into the atmosphere we seek to understand.

To remind Fisher (2009), who describes the collapse of the future as a kind of cancellation of imaginative potential: we live in an endless present, where the future is no longer conceived as rupture, but as more of the same. And yet, paradoxically, it is he who also points to the need to reactivate forbidden desires, to make shimmer what was once silenced. To recover lost futures as a radical listening to the echoes of aborted futures in acts of care, in the persistence of bonds that resist collapse. There’s no time for restoring utopias. The time that remains, then, may not be an ending, but a fold where something can still be done.

French philosopher Georges Didi‐Huberman (2009), in *Survivance des Lucioles* [The Survival of the Fireflies], envisions survival as a form of aesthetic politics. The fireflies—nearly extinct, nearly extinguished—still shine in certain places, at certain times. Their light is fragile, discontinuous, but real. He writes: “The fireflies do not organize themselves into constellations. They appear and disappear, without warning. But one must know how to see them” (p. 24). This intermittent glow is also a form of time. A fidelity to that which still pulses, even if it is not enough to light the way. Analysis, at this point, does not turn on the light: it walks with the fireflies, and perhaps that is the only kind of guide possible in certain moments, the brief glimmer of something that is not yet dead.

This kind of time appears vividly in *To the Lighthouse*, by modernist writer Virginia Woolf (1927/2015)—especially in the chapter “Time Passes”. The family nucleus disappears, the war unfolds, the house dissolves. But time remains as an atmospheric presence, made of dust, of wind, of walls that still listen. Nothing grand occurs, and yet, everything transforms: “thus, slowly, silently, the great tapestry of time is woven without sound” (Woolf, p. 114). The tapestry of time, in this case, is not made of events. It is woven through persistence, attention and listening. The time that remains, in this register, is an Orphic time. It does not bring redemption, but allows existence to hear itself in its minimal duration.

Hilda Hilst (1997), one of the most important poets in Brazil’s literary history, in her singular voice, also touched on this time of remainder: a time of dark matter, of wounded eroticism, of inexplicable yet insistent existence. She writes in “Da Noite” [Of the Night]: “What remains to me, remains./And I cling to it, hungry.” There is hunger in what remains. Desire as survival—desire as a form of creation. And perhaps that is enough to make a world with what is left. Time is no longer a line, but breath. A way of being in time without needing it to lead anywhere. Perhaps the most radical task of analytical listening is not to give meaning to time, but to sustain it when it no longer organizes itself. When the appointed hour does not arrive and the future collapses, what remains is this smaller, fragmented, often dusty time, where breath still happens, where the soul still moves, even without direction. To sustain this time, with attention and presence, is already to transform it: into image, into bond.

Analysis, at this point, becomes not about restoring the timeline of life, but about accompanying the pulse of what survives.

## Conclusion

To listen to psychic suffering that unfolds outside linear time is not a suspension of analysis; it is its most radical act.

This essay traced two temporal figures that emerge in analytical encounters: the hour that never comes, and the time that remains. They do not follow the rhythm of adaptation or resolution, nor do they conform to the metrics of affective capitalism. These are not distortions to be corrected, but symbolic climates through which the soul manifests its presence. The hour that never comes evokes the painful suspension of expectation, the absence of arrival, the event that was meant to happen and never did. The time that remains expresses what still vibrates after collapse, the murmur of meaning where structure has given way. Together, these figures displace the notion of cure as linear progression and invite us to dwell alongside forms of suffering that persist without narrative closure. To engage these temporalities is to risk a different kind of analytic stance, one attuned not to development, but to intensity and reverberation, to the density of atmosphere, rather than to the economy of goals. Listening, in this sense, becomes a work of fidelity to the fragment, the repetition, the residue. It is an Orphic descent into the strata where time folds into image, where presence accumulates by deepening.

And if something sings there, it is not the echo of what was lost, but the strange music of what refuses to end.
